# Assessment of Anxiety- and Depression-like Behaviors and Local Field Potential Changes in a Cryogenic Lesion Model of Traumatic Brain Injury

**DOI:** 10.3390/ijms27020597

**Published:** 2026-01-07

**Authors:** Yeon Hee Yu, Yu Ran Lee, Dae-Kyoon Park, Beomjong Song, Duk-Soo Kim

**Affiliations:** 1 Department of Anatomy, College of Medicine, Soonchunhyang University, Cheonan 31151, Republic of Korea; yyh0220@sch.ac.kr (Y.H.Y.); mdeornfl@sch.ac.kr (D.-K.P.); antares119@gmail.com (B.S.); 2 Department of Anatomy, Convergence of Medicine, Gyeongsang National University, Jinju 52727, Republic of Korea; kbk1999@sch.ac.kr

**Keywords:** traumatic brain injury, emotional phenotypes, hippocampus, local field potentials

## Abstract

Patients with traumatic brain injury (TBI) have an elevated risk of developing chronic psychiatric and behavioral disorders, including impairments in motor function, memory deficits, anxiety, and depression. Although a substantial body of work has addressed the treatment and rehabilitation of sensory, motor, and cognitive symptoms after TBI, there is a relative scarcity of comprehensive behavioral assessments targeting neuropsychiatric manifestations in preclinical models. This study aims to investigate the connections between emotional sequelae after TBI and modifications in local field potentials (LFPs). Following cryogenic lesion-induced TBI, animals exhibited anxiety-like behaviors as assessed by the open field test (*p* < 0.001), light/dark box test (*p* < 0.001), and elevated plus maze test (*p* < 0.01). Depression-like behavior was observed using the forced swim test (*p* < 0.001). LFP analysis demonstrated a marked elevation in neural oscillatory activity associated with anxiety and depression in the contralateral hemisphere relative to the ipsilateral side (*p* < 0.001). These results indicate that the emotional consequences triggered by TBI may be linked to dysregulated synchronous neural activity between the ipsilateral and contralateral hemispheres.

## 1. Introduction

Traumatic brain injury (TBI) refers to sudden damage to the brain resulting from a blow or impact to the head, leading to alterations in neurological function. These injurious forces may involve blunt force trauma, skull penetration, or skull fracture [1]. TBI is classified as mild, moderate, or severe according to clinical parameters, including level of consciousness, presence and duration of amnesia, and other neurological manifestations [2]. Mild TBI represents the most prevalent form, accounting for 80–90% of all TBI cases, whereas severe TBI constitutes approximately 5–20% [3,4]. Furthermore, mild TBI is recognized as a significant risk factor for the later development of neurodegenerative diseases, including Alzheimer’s disease and Parkinson’s disease [3,5]. Individuals who survive TBI are at an increased risk for persistent mental and behavioral disorders. Sensorimotor impairment and learning and memory deficits, as well as anxiety and depression, are commonly observed in cases of mild TBI. Psychiatric complications such as depression and anxiety frequently persist for many years following the initial injury, adversely affecting social integration and overall quality of life. Additionally, the presence of comorbid neuropsychiatric conditions that are associated with emotional and behavioral disturbances may ultimately be linked to an increased risk of suicidal behavior [6]. Existing research has primarily concentrated on therapeutic intervention and rehabilitation for TBI, focusing largely on sensory, motor, and cognitive symptoms, whereas behavioral evaluations for assessing neuropsychiatric manifestations in preclinical models remain less explored. Accordingly, the present study seeks to advance understanding of TBI-associated psychiatric sequelae by examining the association between anxiety- and depression-like behaviors and electrophysiological alterations utilizing a cryogenic lesion TBI model.

## 2. Results

### 2.1. Reduction in Residual Cortical Size Following TBI

**Figure 1:**
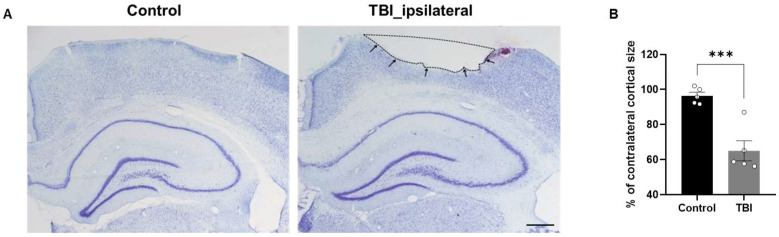
Analysis of remaining cortical size following TBI. (**A**) Representative cresyl violet (Nissl) staining of a control rat and a TBI rat at 7 days post-injury. The black line and arrows demarcate the lesioned cortex. (**B**) Statistical comparison of cortical size between TBI and control rats. Scale bar = 100 μm. Data are presented as means ± standard errors of the mean (SEM). *** *p* < 0.001 by unpaired *t*-test, *n* = 5 per group.

Cresyl violet staining was utilized to assess the residual cortical size in brain sections obtained from control and TBI rats (Figure 1A). The black line delineates the damaged cortical region (Figure 1A). TBI rats exhibited a significant reduction in residual cortical area compared with control rats (*p* < 0.001; *t*-test; Figure 1B).

### 2.2. Decreased Locomotor Activity and Increased Anxiety-Related Behaviors Following TBI

**Figure 2:**
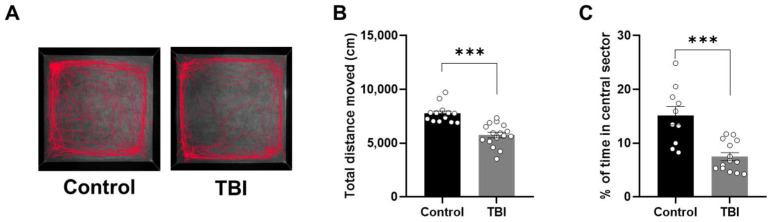
Decreased locomotor activity and increased anxiety-like behavior in the open field test of TBI rats. (**A**) Representative movement traces showing the locomotor activity of control and TBI rats in the open field test. (**B**) Total distance of locomotion by control and TBI rats. (**C**) Time spent in the center sector percentage of control and TBI rats. Data are expressed as mean ± standard error of the mean. *** *p* < 0.001 by unpaired *t*-test (Control, *n* = 13; TBI, *n* = 16).

**Figure 3:**
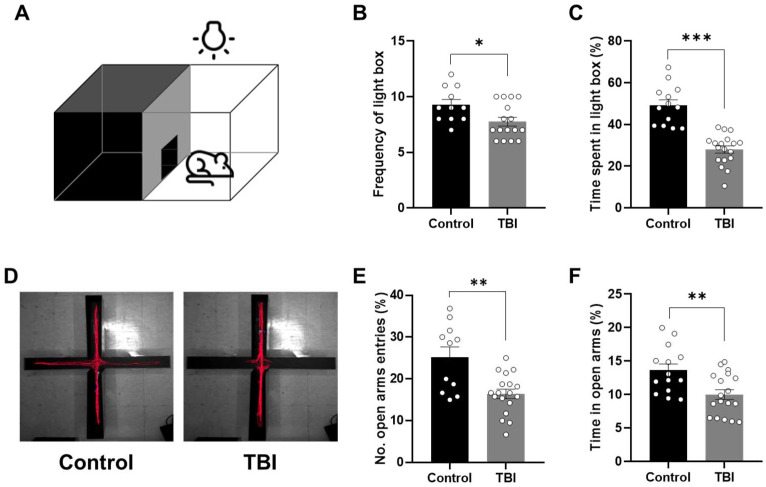
Increased anxiety-like behavior in the light/dark box and elevated plus maze test following TBI in rats. (**A**) Schematic illustration of the light/dark box apparatus, consisting of two compartments. (**B**) Comparison of the frequency of entries into the light box between the control and TBI rats. (**C**) Comparison of the percentage of time spent in the light box. (**D**) Representative movement traces of control and TBI rats in the elevated plus maze. (**E**) Comparison of the percentage of number entries into the open arms. (**F**) Comparison of the percentage of time spent in the open arms. Data are presented as means ± standard errors of the mean. * *p* < 0.05, ** *p* < 0.01, *** *p* < 0.001, by unpaired *t*-test (Control, *n* = 14; TBI, *n* = 18).

At 7 days post-injury, TBI rats exhibited altered locomotor activity compared to control rats in the open field test (Figure 2A). The total distance traveled by TBI rats was significantly reduced relative to control rats (*p* < 0.001; *t*-test; Figure 2B), indicating an association between TBI and reduced locomotor activity. To assess the impact of TBI on anxiety-related behaviors, we conducted three established anxiety-related behavioral assays in TBI rats. In the open field test, TBI rats spent significantly less time in the center sector compared to controls (*p* < 0.01; *t*-test; Figure 2C). In the light/dark box test (Figure 3A), TBI rats showed a significant reduction in the number of entries into the light box in comparison to control rats (*p* < 0.05; *t*-test; Figure 3B). Furthermore, TBI rats also spent less time in the light box relative to controls (*p* < 0.001; *t*-test; Figure 3C). The elevated plus maze, consisting of a plus-shaped apparatus with two open and two closed arms, was used to evaluate anxiety-like phenotypes in TBI rats (Figure 3D). A significant reduction in the percentage of open arm entries was observed in TBI rats relative to controls (*p* < 0.01; *t*-test; Figure 3E). Similarly, the percentage of time spent in the open arms was significantly lower in TBI rats than in controls (*p* < 0.01; *t*-test; Figure 3F). Collectively, these results indicate that TBI is associated with alterations in anxiety-like behaviors in rats.

### 2.3. Increased Levels of Depression-Related Behaviors Following TBI

**Figure 4:**
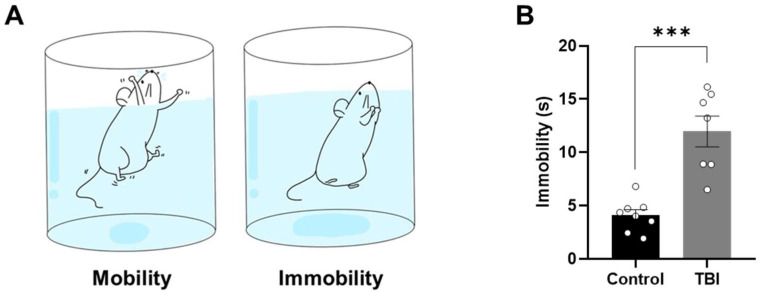
Depression-like behavior increased in forced swim test following TBI in rats. (**A**) Schematic illustration of the forced swim test. (**B**) Comparison of immobility time during the forced swim test between control and TBI rats. Data are presented as means ± standard errors of the mean. *** *p* < 0.001 by unpaired *t*-test (Control, *n* = 7; TBI, *n* = 11).

To assess depression-like behaviors associated with TBI, a forced swim test was performed in TBI rats using validated procedures described previously [7,8]. In the forced swim test (Figure 4A), TBI rats exhibited increased immobility time compared to controls (*p* < 0.05; *t*-test; Figure 4B).

### 2.4. Representative Profiles of LFP After TBI

**Figure 5:**
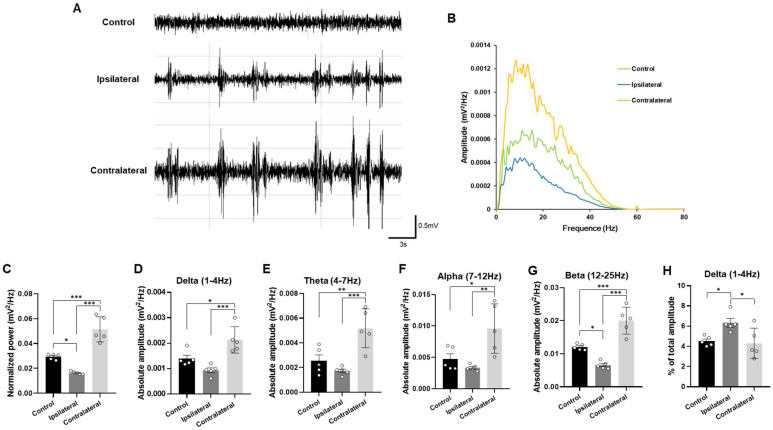
Enhanced oscillatory activity in the contralateral hippocampus of TBI rats. (**A**) Representative raw local field potential (LFP) traces recorded from the CA1 region of the hippocampus under urethane-induced anesthesia. (**B**) Power spectral analysis of LFPs recorded from the CA1 region in control rats and from the ipsilateral and contralateral hippocampal CA1 region in TBI rats. (**C**–**H**) Comparison of representative power spectral analyses among control rats, the ipsilateral hippocampus of TBI rats, and the contralateral hippocampus of TBI rats. (**C**) Comparison of normalized LFP power across groups. (**D**) Comparison of absolute delta band power across groups. (**E**) Comparison of absolute theta band power across groups. (**F**) Comparison of absolute alpha band power across groups. (**G**) Comparison of absolute beta band power across groups. (**H**) Comparison of delta band power as a proportion of total power. Data are reported as means ± standard errors of the mean. * *p* < 0.05, ** *p* < 0.01, *** *p* < 0.001 by unpaired *t*-test (Control, *n* = 5; TBI, *n* = 5).

To examine the association between alterations in 4–25 Hz oscillatory activity in the hippocampus and emotional states such as anxiety and depression in a TBI rat model, LFPs were recorded from the CA1 region of the hippocampus following TBI induction. Recordings were obtained separately from the ipsilateral and contralateral hemispheres. Compared to controls, TBI rats exhibited epileptiform discharges in the CA1 region, marked by irregular sharp waves and intermittent large-amplitude spikes with multiple spikes (Figure 5A). Importantly, the power spectral density of LFPs was reduced on the ipsilateral side and elevated on the contralateral side in TBI rats relative to controls (Figure 5B). Consistently, normalized LFP power was similarly decreased on the ipsilateral side and increased on the contralateral side in TBI rats versus controls (ipsilateral side, *p* < 0.05; contralateral side, *p* < 0.001; one-way ANOVA; Figure 5C). The absolute amplitude in the delta frequency band was also higher on the contralateral side compared to control rats (*p* < 0.05; one-way ANOVA; Figure 5D). Furthermore, synchronous neuronal activity in the theta (4–7 Hz), alpha (7–12 Hz), and beta (12–25 Hz) bands on the contralateral side was significantly increased compared to controls (theta, *p* < 0.01; alpha, *p* < 0.05; beta, *p* < 0.001; one-way ANOVA; Figure 5E–G). These frequency bands have been previously reported to be associated with anxiety- and depression-related behaviors [7,9]. Of note, delta rhythm is a characteristic LFP pattern of the epileptic hippocampus [10,11], and on the ipsilateral side, the percentage of delta power was significantly higher compared to control rats (*p* < 0.05; one-way ANOVA; Figure 5H).

## 3. Discussion

The etiology of psychiatric disorders and behavioral alterations in TBI models remains poorly understood, and it is unclear whether psychiatric sequelae following TBI represent a cause or a consequence of functional impairments. Accordingly, the present study sought to characterize the development of emotional sequelae associated with TBI.

Depression and anxiety are frequently observed psychiatric sequelae in patients with TBI, with a reported prevalence exceeding 70% [12]. Consistent with these clinical observations, our TBI model exhibited comparable behavioral alterations. Previous studies have identified asymmetric theta rhythm and theta-frequency synchronization as potential biomarkers of depression and anxiety in humans [9,13]. Furthermore, animal studies indicate that theta rhythms become more prominent during fear-related and anxiety-like states, supporting a close association between theta activity and emotional processes related to depression and anxiety [14,15,16]. The hippocampus is a major generator of theta oscillations projecting to the frontal cortex, and alterations in hippocampal theta oscillations and temporal lobe alpha activity have been reported in patients exhibiting depressive symptoms [9,17,18]. Consistent with previous studies, our findings indicate that heightened theta and alpha activity in the hippocampus on the contralateral side may be associated with oscillatory patterns previously reported in relation to depression and anxiety symptoms [7,19]. Clinical investigations into depression have documented a general decline in alpha activity; however, increased alpha power has been noted in the ventral prefrontal cortex as well as in posterior regions. Notably, a significant elevation of posterior alpha power in the left hemisphere relative to the right has been reported, which has been interpreted as reflecting reduced cortical activity in the left posterior region [20,21]. In addition, several studies have shown that individuals with depression exhibit greater alpha activity in the left frontal lobe compared to the right [22]. We hypothesize that the elevated alpha power observed in the contralateral (left) hippocampus may reflect neural states associated with depressive phenotypes in our TBI model. In those diagnosed with major depressive disorder, increased beta power has been detected both at rest and during movement [23], though diminished activity in the frontal cortex and dorsal anterior cingulate cortex has also been observed [20,24]. The association between alpha–beta coupling and depression has attracted growing attention, and the alpha/beta ratio has been proposed as a potential measure for characterizing depressive features [20,25]. However, despite increased beta power after TBI in our animal model, the alpha/beta ratio calculated from hippocampal depth LFPs did not correlate significantly with either the manifestation or severity of depressive symptoms (see Supplementary Figure S1).

Our results revealed increased delta-band power on the ipsilateral side, a well-established electrophysiological feature associated with epileptic activity [18,19]. Additionally, we observed a decline in LFP power in the ipsilateral hemisphere, resulting in overall reduced absolute amplitude, whereas the contralateral hemisphere showed increased normalized power and absolute amplitude. The enhanced neural activity observed in the contralateral side may represent a compensatory response to reduced activity in the ipsilateral hemisphere and may be associated with vulnerability to depression- and anxiety-like behaviors.

In our study, the cryogenic lesion model induces a highly focal cortical injury by applying localized cooling to a restricted cortical area, resulting in well-demarcated and highly reproducible lesions. Accordingly, this model is particularly well suited for investigating neurophysiological and behavioral changes associated with focal cortical damage. In addition, previous studies have demonstrated that the cryogenic lesion model can be used to examine long-term neurodegenerative changes resembling late complications of human traumatic brain injury, including global brain atrophy, cognitive impairment, and behavioral abnormalities [26]. In contrast, the fluid percussion injury (FPI) model involves performing a craniotomy to expose a portion of the dura mater, followed by the delivery of a fluid pressure pulse to induce injury. Although the precise location and extent of the lesion are more difficult to control, the FPI model produces pathological features resembling clinical concussion and diffuse axonal injury, making it particularly suitable for studying diffuse brain injury and widespread network disruption [27]. The controlled cortical impact (CCI) model induces a relatively focal cortical injury through a mechanical impact, while also producing secondary injury and diffuse pathology in the perilesional regions [26]. This model reproduces pathological features similar to cortical contusions observed in clinical TBI. Consequently, both the CCI and FPI models can be used to induce mild, moderate, or severe traumatic brain injury depending on the injury parameters. Taken together, integrating findings from these three complementary experimental models may help overcome the translational limitations of individual approaches and provide a more comprehensive understanding of the pathophysiology of traumatic brain injury.

## 4. Materials and Methods

### 4.1. Experimental Animals

All experiments utilized male Sprague Dawley rats (8 weeks old, 270–290 g) obtained from Soonchunhyang University Laboratory Animal Center (Cheonan, Republic of Korea). Animals had unrestricted access to a commercial diet and water, and were maintained under controlled environmental conditions (12:12 light/dark cycle, 22 ± 2 °C, 55 ± 5% humidity). The study protocols were approved by the Animal Laboratory Management Committee of Soonchunhyang University (permit number SCH23-0025). Every measure was taken to minimize animal suffering and reduce the number of animals used throughout the experimental process.

### 4.2. TBI Induction

8-week-old male SD rats (270–290 g) were anesthetized via intraperitoneal injection (i.p.) of ketamine (90 mg/kg) and xylazine (4 mg/kg). Following a midline scalp incision made under a stereotaxic frame, the frontoparietal skull was exposed, and a cryogenic lesion was induced on the right frontoparietal cortex (unilateral lesions). To produce the lesion, a cone-shaped copper cylinder with a 2 mm tip diameter was cooled using acetone and dry ice. Previous studies using the same cryogenic setup have demonstrated that the probe tip reliably reaches around −78 °C under these conditions [28]. The chilled probe was stereotaxically positioned in direct contact with the exposed skull at coordinates relative to bregma—1.5 mm posterior and 1.5 mm lateral—and maintained in place for 1 min [29,30]. Sham-operated animals underwent the same surgical procedure without cooling of the probe. Subsequently, lesion volume was histologically quantified using Nissl staining.

### 4.3. Behavioral Tests

**Figure 6:**
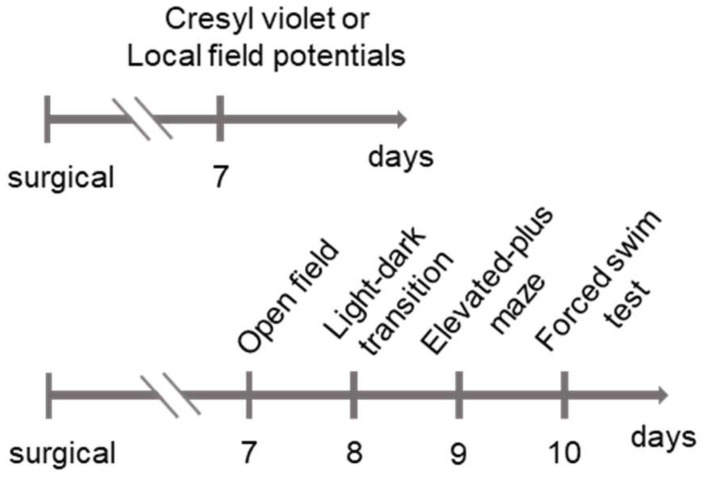
A representative schematic illustrating the timelines for behavioral tests, histological analysis, and local field potential recordings. Cresyl violet staining was performed 7 days after surgery. Behavioral assessments were conducted sequentially, starting with the least stressful tests to minimize stress-related confounding effects.

All experimental animals underwent serial behavioral assessments for locomotor activity, as well as anxiety- and depression-related behaviors, at 7 days post-injury. To minimize environmental novelty effects, all experimental animals were habituated with a 60 Hz white noise exposure for 30 min to 1 h prior to testing for each behavioral assay. Testing was conducted between 9 a.m. and 5 p.m. using adult rats. Behavioral data were captured and analyzed using a PC-based video analysis system equipped with the automated tracking software Noldus EthoVision XT 14 (Wageningen, The Netherlands). Emotional behavioral tests, histological staining, and LFP recordings were performed following the procedures outlined in Figure 6.

#### 4.3.1. Open-Field Test

The open field test (OFT) leverages the natural tendency of experimental animals to explore new environments. This protocol provides measures of both general motor activity and anxiety-related behaviors [17]. Animals were placed in the center of a rectangular open field (60 × 60 cm) within an apparatus measuring 60 × 60 × 40 cm under diffused lighting. During a 30 min session, total distance traveled was used to assess locomotor activity, while time spent in the center area was used as an index of anxiety.

#### 4.3.2. Light–Dark Transition Test

This test assesses anxiety-like behavior based on the animal’s innate preference for darkness and avoidance of brightly lit areas. The apparatus is separated into two compartments (45 × 27 × 27 cm; length, width, height) connected by small holes. The light compartment comprised 3/5 of the total space, while the dark compartment covered the remaining 2/5, divided by black slats containing holes for passage. Illumination in the bright compartment was maintained at 300 lux, and 0–1 lux in the dark compartment. Experimental animals were introduced into the light chamber and allowed to explore freely while behavior was videotaped for 5 min. Anxiety-like behavior was quantified by measuring the time spent in the lighted area and the number of transitions between compartments [31,32].

#### 4.3.3. Elevated-Plus Maze Test

The elevated plus maze (EPM) is a standard behavioral paradigm for evaluating anxiolytic or anxiogenic responses in rodents [33]. The plus-shaped apparatus (elevated 60 cm above the floor) consisted of two open and two closed arms. Each arm of the maze had the following dimensions: open arms (50 × 10 × 1 cm; length, width, height) and closed arms (50 × 10 × 30 cm; length, width, height). Lighting in the center area of the maze was maintained at 200 lux. The animal was placed in the central region, oriented toward one of the open arms, and monitored for 5 min. The number of open arm entries and the total duration spent in the open arms were recorded [31].

#### 4.3.4. Forced Swim Test

A forced swim test (FS) was conducted to assess depression-like behavior [7,34]. Each experimental animal was placed individually in a plastic cylinder (50 cm height, 30 cm diameter) filled with water (23 ± 3 °C) and monitored for 5 min. The duration for which the experimental animals remained immobile was recorded. Immobility time is interpreted as an indicator of depression-like behavior. After the test, animals were removed from the water, thoroughly dried, and returned to their cages.

### 4.4. Local Field Potentials (LFPs)

Animals received intraperitoneal injections of urethane (1.5 g/kg) for anesthesia and were subsequently positioned in a stereotaxic apparatus. Burr holes were made in the skull to insert the electrodes. LFPs were acquired using a tungsten parylene-coated electrode (0.005-inch outer diameter; A-M Systems, Sequim, WA, USA). The electrode was positioned according to these coordinates (relative to CA1): 3.8 mm posterior to bregma, 2.5 mm lateral to the midline, and 2.9 mm in depth. Recordings were made with a QP511 AC amplifier (0.1–3000 Hz bandpass, GRASS Technologies, West Warwick, RI, USA). Signals were digitized at 5 kHz and used to obtain 2 h of baseline data. All recordings from single channels were captured using Axoscope 10.2 software (Axon Instruments, Union City, CA, USA). Subsequent analyses of these traces were performed using Clampfit 10.2 software (Axon Instruments, Union City, CA, USA). For frequency-domain analysis, LFP activity in the 1–25 Hz range was analyzed based on previous studies, focusing on frequency bands commonly associated with disease states and emotional processing: δ (1–4 Hz), θ (4–7 Hz), α (7–12 Hz), and β (12–25 Hz) [35,36]. Amplitude spectra of normalized power were derived for each frequency, and root mean square (RMS) values were calculated to estimate spectral power (mV^2^) in 1 Hz bins for each electrode. Spectral power data were averaged across all epochs within single baseline session and are presented as mV^2^/Hz. For each animal, fast Fourier transform (FFT) was performed on the epochs for all electrodes at a frequency resolution of 0.61 Hz, and the resulting spectra were averaged. Although Hamming windows were applied to minimize edge-related artifacts during FFT analysis, the use of non-overlapping windows may still lead to spectral leakage, particularly in non-stationary LFP signals, in which power from a given frequency component spreads into adjacent frequency bins.

### 4.5. Tissue Processing and Cresyl Violet (CV) Staining

Anesthetized animals (urethane, 1.5 g/kg, i.p.) were perfused transcardially with PBS and subsequently fixed with 4% PFA in 0.1 M PB (pH 7.4). Brains were extracted and postfixed in the same fixative solution for 4 h at room temperature. Brain tissue was permeabilized in 30% sucrose overnight and stored at 4 °C. The entire brain was then frozen and cut into 30 μm sections using a cryostat, with consecutive sections transferred to a 6-well plate containing PBS. For analysis of the residual cortex after TBI, brain sections were mounted onto gelatin-coated slides and each slide was stained with cresyl violet acetate (Sigma, St. Louis, MO, USA). Images were captured using a DP72 digital camera in conjunction with DP2-BSW microscope digital camera software (Olympus, Tokyo, Japan) to quantify the size of the residual cortex post-TBI.

### 4.6. Statistical Analysis

Quantification and statistical analysis of data were performed following procedures described in a previous study, with minor modifications [7]. Data are presented as mean ± standard error of the mean (SEM). Statistical analyses were conducted using GraphPad Prism 8 (GraphPad Software, San Diego, CA, USA). Behavioral and histological data obtained from quantitative measurements were analyzed using Student’s *t*-test to assess statistical significance. LFPs data were analyzed using one-way analysis of variance (ANOVA) to evaluate statistical significance. Bonferroni’s post hoc test was applied for multiple comparisons. Differences were considered statistically significant at *p*-values < 0.05, <0.01, or <0.001.

## 5. Conclusions

In summary, this study indicates that the emotional phenotypes identified after TBI may be linked to increased synchronous neuronal activity related to depression and anxiety in the contralateral side. These findings offer preclinical evidence supporting the contralateral side as a potential target for neuro-feedback interventions addressing psychiatric sequelae post-TBI. Nevertheless, the present study is limited by the use of anesthetized, resting-state LFP recordings and the absence of significant differences in cross-frequency metrics, which may have constrained the detection of more dynamic neuronal interactions. In addition, the relatively small sample size, the exclusive use of male rats, and the assessment of behavioral and electrophysiological outcomes at a single subacute time point may limit the generalizability of our findings. Additional studies employing awake recording paradigms, longitudinal designs across multiple post-injury time points, and larger, sex-balanced sample sizes will be necessary to more precisely characterize the neurological and physiological distinctions between the ipsilateral and contralateral sides. Moreover, further validation is required to strengthen the translational relevance of this preclinical model to TBI-related neuropsychiatric systems.

## Data Availability

The original contributions presented in this study are included in the article/Supplementary Materials. Further inquiries can be directed to the corresponding author.

## Supplementary Materials

The following supporting information can be downloaded at: https://www.mdpi.com/article/10.3390/ijms27020597/s1.
