# Supplementary material for: Assessment of Anxiety- and Depression-like Behaviors and Local Field Potential Changes in a Cryogenic Lesion Model of Traumatic Brain Injury
Source: Int J Mol Sci. 2026 Jan 7;27(2):597. doi: 10.3390/ijms27020597 (PMC12841474; doi:10.3390/ijms27020597)
Supplement: Supplementary file 1 [file ijms-27-00597-s001.zip › ijms-4019998-supplementary.pdf]

Supplementary Figure

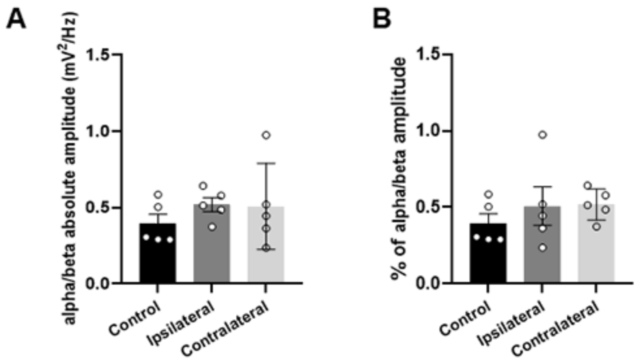

Supplementary Figure S1. No significant correlation was observed between the alpha and beta ratios of TBI rats. (A) Comparison of absolute alpha/beta power ratios among control rats, the ipsilateral hippocampus of TBI rats, and the contralateral hippocampus of TBI rats. (B) Comparison of the normalized alpha/beta power ratio, expressed as a proportion of total power, among control rats, the ipsilateral hippocampus of TBI rats, and the contralateral hippocampus of TBI rats. Data are reported as means ± standard errors of the mean. (Control, n = 5; TBI, n = 5).
